# LPS Auto-Calibration Algorithm with Predetermination of Optimal Zones

**DOI:** 10.3390/s111110398

**Published:** 2011-10-31

**Authors:** Francisco Daniel Ruiz, Jesús Ureña, Juan C. García, Ana Jiménez, Álvaro Hernández, Juan J. García

**Affiliations:** Electronics Department, University of Alcalá de Henares, Escuela Politécnica. Ctra. Madrid-Barcelona, Km. 33,600, 28871 Alcalá de Henares, Spain; E-Mails: urena@depeca.uah.es (J.U.); jcarlos@depeca.uah.es (J.C.G.); ajimenez@depeca.uah.es (A.J.); alvaro@depeca.uah.es (A.H.); jesus@depeca.uah.es (J.J.G.)

**Keywords:** LPS, autocalibration, spherical and hyperbolic trilateration, optimal test points

## Abstract

Accurate coordinates for active beacons placed in the environment are required in Local Positioning Systems (LPS). These coordinates and the distances (or differences of distances) measured between the beacons and the mobile node to be localized are inputs to most trilateration algorithms. As a first approximation, such coordinates are obtained by means of manual measurements (a time-consuming and non-flexible method), or by using a calibration algorithm (*i.e*., automatic determination of beacon coordinates from *ad hoc* measurements). This paper presents a method to calibrate the beacons’ positions in a LPS using a mobile receiver. The method has been developed for both, spherical and hyperbolic trilateration. The location of only three test points must be known *a priori*, while the position of the other test points can be unknown. Furthermore, the paper describes a procedure to estimate the optimal positions, or approximate areas in the coverage zone, where the test-points necessary to calibrate the ultrasonic LPS should be placed. Simulation and experimental results show the improvement achieved when these optimal test-points are used instead of randomly selected ones.

## 1. Introduction

Research in Local Positioning Systems (LPS) is an area of great interest due to the large number of applications supported by this kind of system. Outdoor localization is often carried out using GPS, but, indoors, it is difficult to use because GPS signals become weak. To solve the indoor localization problem, several methods have been proposed [1]. One of the most used solutions uses several beacons placed in the environment in order to emulate the GPS satellites [2,3]. In these systems, in order to locate an object it is necessary to know the positions of the beacons. Usually the position of the beacons with respect to a reference coordinate system is measured by hand. This results in low portability and adaptability since the calibration process requires time and effort.

Thanks to auto-calibration techniques, a system can automatically compute the position of beacons by taking measurements from specific known positions. Several previous works have addressed this problem. In [4] a spherical auto-calibration system based on inverse positioning is proposed. The positions of beacons are obtained by taking measurements from several known locations and by applying the positioning algorithm in an inverse way. In that work all the beacon signals must be detected from the known measurement locations. In order to improve the previous system, without the need for detecting all the beacons at the same time, in [5] several reference systems are used to unify them afterwards, by using those beacons common to measurements carried out from different reference systems. In [6] a recurrent spherical auto-calibration algorithm is proposed by using a non-linear square minimization and a fitness function to find the most accurate solution. In this method the measured distance data is divided in several subsets and in each group the algorithm calculates one hundred of low precision solutions, using after a fitness function to select the best six solutions. Finally, it uses a refinement process to obtain the best solution. In [7] a similar algorithm for hyperbolic trilateration is proposed. These methods are developed for a great amount of data (high redundancy) and a high uncertainty in the measurements, so they have problems of convergence and need sophisticated refinement techniques.

This paper describes a new algorithm for the calibration of a LPS, allowing the system to calculate the positions of beacons. In this case it is assumed that the set of measurements has less uncertainty [8] than the aforementioned ones, and so the proposed process is easier to implement and guarantees the convergence in most practical situations. This method only requires knowing the location of three measurement points to find the beacons, whereas the other test points can be placed at unknown positions. Additionally, taking advantage of this fact, a method is proposed to obtain the optimal zones to make the measurements during the auto-calibration process, as an additional contribution of this work not found in the previous algorithms. The optimal placement of the beacons to cover a determined positioning area has been previously considered in the literature [9]; the approach here is in the inverse way: a coarse estimation of the positions of the beacons is known and the goal is to determine the optimal zones to place the test points in the calibration process.

This document is divided as follows: in Section 2 the proposed algorithms to perform the auto-calibration are detailed; the localization of the best areas to place the test points at is analyzed in Section 3; simulation and experimental results are shown in Sections 4 and 5, respectively; and finally the conclusions are discussed in Section 6.

## 2. Proposed Auto-Calibration Algorithm

Two methods are proposed in this section. The first algorithm is suitable for systems based on spherical trilateration, whereas the second one is for hyperbolic trilateration. Furthermore, this last case does not require synchronization between beacons and the mobile node.

**Figure 1.**
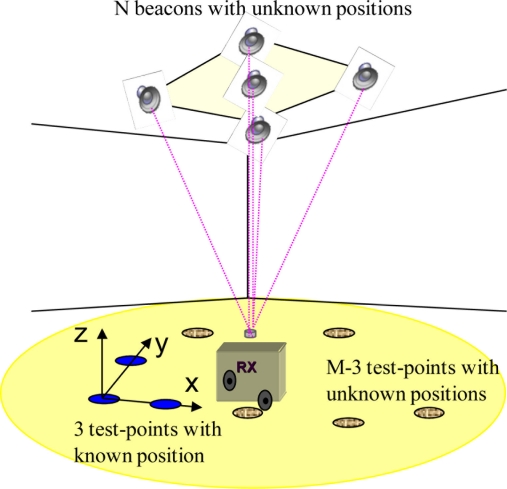
Representation of the LPS to be calibrated.

As has been mentioned before, to calculate the coordinates of beacons, it is necessary to measure the distances to beacons from several test points. The location of these test points is also unknown to the algorithm except for the position of three that fix the coordinate system. Thus, the initial information in both algorithms is (see Figure 1):

- A vector that contains the “initial approximate position” **iap** for every unknown of the system: the position of the *N* beacons and the position of the *M*-3 unknown test-point locations: ***iap*** = (*b̠_x_*_1_, *b̠_y_*_1_, *b̠_z_*_1_,⋯, *b̠_zN_*, *b̠_yN_*, *b̠_zN_*, *x̂*_4_, *ŷ*_4_,⋯,*x̂_M_*, *ŷ_M_*).

The height (z-component) of the test points is known (*i.e*., *z* = 0).

- The distances between the test points and the beacons: in the case of spherical positioning it refers to the absolute distances to the beacons; whereas, in the case of hyperbolic positioning, it refers to the differences between the distances to each beacon and the distance to one of the beacons considered the reference.
- The locations of the three known test points to avoid ambiguities in the solution: [***X***_1_, ***X***_2_, ***X***_3_].

### 2.1. Auto-Calibration with Spherical Trilateration

The absolute distance between the beacon *i* with coordinates (*b_xi_*, *b_yi_*, *b_zi_*) and the test point *m* with coordinates (*x_m_*, *y_m_*, *z_m_*) is defined as:

(1) $$r_{\mathrm{im}}=\sqrt{{\left(b_{\mathrm{xi}}-x_{m}\right)}^{2}+{\left(b_{\mathrm{yi}}-y_{m}\right)}^{2}+{\left(b_{\mathrm{zi}}-z_{m}\right)}^{2}}$$

And for the estimations (*b̠_xi_*, *b̠_yi_*, *b̠_zi_*) and (*x̠_m_*, *ŷ_m_*, *z̠_m_*), the estimated distance is:

(2) $${\hat{r}}_{\mathrm{im}}=\sqrt{{\left({\hat{b}}_{\mathrm{xi}}-{\hat{x}}_{m}\right)}^{2}+{\left({\hat{b}}_{\mathrm{yi}}-{\hat{y}}_{m}\right)}^{2}+{\left({\hat{b}}_{\mathrm{zi}}-{\hat{z}}_{m}\right)}^{2}}$$

The proposed method uses the Gauss-Newton algorithm to minimize the sum of the quadratic errors that exist between the measured distances and the distances obtained from the estimated points according to the following expression:

(3) $$F=\sum_{i=1}^{N}\sum_{m=4}^{M}{\left({\hat{r}}_{\mathrm{im}}-r_{\mathrm{im}}\right)}^{2}=\sum_{i=1}^{N}\sum_{m=4}^{M}{\left[f_{\mathrm{im}}\right]}^{2}$$

where:

(4) $$f_{\mathrm{im}}=\sqrt{{\left({\hat{b}}_{\mathrm{xi}}-{\hat{x}}_{m}\right)}^{2}+{\left({\hat{b}}_{\mathrm{yi}}-{\hat{y}}_{m}\right)}^{2}+{\left({\hat{b}}_{\mathrm{zi}}-{\hat{z}}_{m}\right)}^{2}}-r_{\mathrm{im}}$$

Differentiating *f_im_* with respect to all the unknowns of the system and reorganizing the terms of the form ***A*** · Δ ***X*** =***B***:

(5) $$\begin{array}{c} A=\left(\begin{array}{cccccc} \frac{\partial f_{11}}{{\partial \hat{b}}_{x1}} & \ldots & \frac{{\partial f}_{11}}{{\partial \hat{b}}_{\mathrm{zN}}} & \frac{{\partial f}_{11}}{{\partial \hat{x}}_{4}} & \cdots & \frac{{\partial f}_{11}}{{\partial \hat{y}}_{M}}\\ \frac{{\partial f}_{12}}{{\partial \hat{b}}_{x1}} & \cdots & \frac{{\partial f}_{12}}{{\partial \hat{b}}_{\mathrm{zN}}} & \frac{{\partial f}_{12}}{{\partial \hat{x}}_{4}} & \cdots & \frac{{\partial f}_{12}}{{\partial \hat{y}}_{M}}\\ \vdots & \ddots & \vdots & \vdots & \ddots & \vdots \\ \frac{{\partial f}_{\mathrm{NM}}}{{\partial \hat{b}}_{x1}} & \ldots & \frac{{\partial f}_{\mathrm{NM}}}{{\partial \hat{b}}_{\mathrm{zN}}} & \frac{{\partial f}_{\mathrm{NM}}}{{\partial \hat{x}}_{4}} & \cdots & \frac{{\partial f}_{\mathrm{NM}}}{{\partial \hat{y}}_{M}} \end{array}\right)\\ \Delta X={\left[{\hat{b}}_{x1},{\hat{b}}_{y1},{\hat{b}}_{z1},\cdots ,{\hat{b}}_{\mathrm{yN}},{\hat{b}}_{\mathrm{zN}},{\hat{x}}_{4},{\hat{y}}_{4},\cdots ,{\hat{x}}_{M},{\hat{y}}_{M}\right]}^{T}\\ B={\left[f_{11}\cdots f_{\mathrm{NM}}\right]}^{T} \end{array}$$

where:

(6) $$\frac{{\partial f}_{\mathrm{im}}}{{\partial \hat{b}}_{\mathrm{ai}}}=\frac{{\hat{b}}_{\mathrm{ai}}-{\hat{a}}_{m}}{{\hat{r}}_{\mathrm{im}}};\frac{{\partial f}_{\mathrm{im}}}{{\partial \hat{a}}_{m}}=\frac{{\hat{a}}_{m}-{\hat{b}}_{\mathrm{ai}}}{{\hat{r}}_{\mathrm{im}}}\;\mathrm{with}\left\{ \begin{array}{l} a=x,y,z\\ i=1\ldots N\\ m=4\ldots M \end{array}\right.$$

Solving the system by Least Mean Squares, the following expression is obtained:

(7) $$\Delta X={\left(A^{T}\cdot A\right)}^{-1}\cdot A^{T}\cdot B$$

After obtaining the increment of the position vector Δ ***X***, the estimated position vector is updated:

(8) $${\mathrm{iap}}_{k+1}={\mathrm{iap}}_{k}-\Delta X_{k}$$

This process is repeated until the increment of the position is lower than a pre-determined threshold.

### 2.2. Auto-Calibration with Hyperbolic Trilateration

In this case, instead of the distance between the test point and the beacon, the difference in distance Δ r between the test-point and the beacon is measured and the test-point and a beacon considered as reference.

Defining the increment of distance between the beacon *i* ≠ 1 and the test-point *m* with respect to the reference beacon (*i* = 1) for the test-point *m* of the form:

(9) $$\begin{array}{c} \Delta r_{\mathrm{im}}=\sqrt{{\left(b_{\mathrm{xi}}-x_{m}\right)}^{2}+{\left(b_{\mathrm{yi}}-y_{m}\right)}^{2}+{\left(b_{\mathrm{zi}}-z_{m}\right)}^{2}}-\sqrt{{\left(b_{x1}-x_{m}\right)}^{2}+{\left(b_{y1}-y_{m}\right)}^{2}+{\left(b_{z1}-z_{m}\right)}^{2}}\\ \mathrm{with}\;i\;=2\ldots N\;\mathrm{and}\;m=4\ldots M \end{array}$$

Also, the increment of distance with the estimated points is:

(10) $$\begin{array}{c} \Delta {\hat{r}}_{\mathrm{im}}=\sqrt{{\left({\hat{b}}_{\mathrm{xi}}-{\hat{x}}_{m}\right)}^{2}+{\left({\hat{b}}_{\mathrm{yi}}-{\hat{y}}_{m}\right)}^{2}+{\left({\hat{b}}_{\mathrm{zi}}-{\hat{z}}_{m}\right)}^{2}}-\sqrt{{\left({\hat{b}}_{x1}-{\hat{x}}_{m}\right)}^{2}+{\left({\hat{b}}_{y1}-{\hat{y}}_{m}\right)}^{2}+{\left({\hat{b}}_{z1}-{\hat{z}}_{m}\right)}^{2}}\\ \mathrm{with}\;\;i\;=2\ldots N\;\mathrm{and}\;m=4\ldots M \end{array}$$

Using the same strategy as in the spherical case, the mean quadratic error between the increments of measured and estimated distances are iteratively minimized with the following expression:

(11) $$G=\sum_{i=2}^{N}\sum_{m=4}^{M}{\left(\Delta \;{\hat{r}}_{\mathrm{im}}-\Delta r_{\mathrm{im}}\right)}^{2}=\sum_{i=2}^{N}\sum_{m=4}^{M}{\left[g_{\mathrm{im}}\right]}^{2}$$

where:

(12) $$\begin{array}{l} g_{\mathrm{im}}=(\sqrt{{\left({\hat{b}}_{\mathrm{xi}}-{\hat{x}}_{m}\right)}^{2}+{\left({\hat{b}}_{\mathrm{yi}}-{\hat{y}}_{m}\right)}^{2}+{\left({\hat{b}}_{\mathrm{zi}}-{\hat{z}}_{m}\right)}^{2}}\\ \;\;\;\;\;\;\;\;\;\;\;\;\;\;\;\;\;\;\;-\sqrt{{\left({\hat{b}}_{x1}-{\hat{x}}_{m}\right)}^{2}+{\left({\hat{b}}_{y1}-{\hat{y}}_{m}\right)}^{2}+{\left({\hat{b}}_{z1}-{\hat{z}}_{m}\right)}^{2}})-\Delta \;r_{\mathrm{im}} \end{array}$$

Differentiating with respect to all the unknowns and regrouping the terms, as in the spherical case, it is obtained:

(13) $$\begin{array}{c} A=\left(\begin{array}{cccccc} \frac{{\partial g}_{21}}{{\partial \hat{b}}_{x1}} & \ldots & \frac{{\partial g}_{21}}{{\partial \hat{b}}_{\mathrm{zN}}} & \frac{{\partial g}_{21}}{{\partial \hat{x}}_{4}} & \cdots & \frac{{\partial g}_{21}}{{\partial \hat{y}}_{M}}\\ \frac{{\partial g}_{22}}{{\partial \hat{b}}_{x1}} & \cdots & \frac{{\partial g}_{22}}{{\partial \hat{b}}_{\mathrm{zN}}} & \frac{{\partial g}_{22}}{{\partial \hat{x}}_{4}} & \cdots & \frac{{\partial g}_{22}}{{\partial \hat{y}}_{M}}\\ \vdots & \ddots & \vdots & \vdots & \ddots & \vdots \\ \frac{{\partial g}_{\mathrm{NM}}}{{\partial \hat{b}}_{x1}} & \ldots & \frac{{\partial g}_{\mathrm{NM}}}{{\partial \hat{b}}_{\mathrm{zN}}} & \frac{{\partial g}_{\mathrm{NM}}}{{\partial \hat{x}}_{4}} & \cdots & \frac{{\partial g}_{\mathrm{NM}}}{{\partial \hat{y}}_{M}} \end{array}\right)\\ \Delta X={\left[{\hat{b}}_{x1},{\hat{b}}_{y1},{\hat{b}}_{z1},\cdots ,{\hat{b}}_{\mathrm{yN}},{\hat{b}}_{\mathrm{zN}},{\hat{x}}_{4},{\hat{y}}_{4},\cdots ,{\hat{x}}_{M},{\hat{y}}_{M}\right]}^{T}\\ B={\left[g_{21}\cdots g_{\mathrm{NM}}\right]}^{T} \end{array}$$

where:

(14) $$\begin{array}{c} \frac{{\partial \;g}_{\mathrm{im}}}{{\partial \hat{b}}_{a1}}=\frac{{\hat{a}}_{m}-{\hat{b}}_{a1}}{{\hat{r}}_{\mathrm{im}}};\frac{{\partial \;f}_{\mathrm{im}}}{{\partial \hat{b}}_{\mathrm{ai}}}=\frac{{\hat{b}}_{\mathrm{ai}}-{\hat{a}}_{m}}{{\hat{r}}_{\mathrm{im}}}\\ \frac{{\partial \;g}_{\mathrm{im}}}{{\partial \;\hat{a}}_{m}}=\frac{{\hat{a}}_{m}-{\hat{b}}_{\mathrm{ai}}}{{\hat{r}}_{\mathrm{im}}};-\frac{{\hat{a}}_{m}-{\hat{b}}_{\mathrm{ai}}}{{\hat{r}}_{\mathrm{im}}}\;\;\;\;\mathrm{with}\left\{ \begin{array}{l} a=x,y,z\\ i=2\ldots N\\ m=4\ldots M \end{array}\right. \end{array}$$

Solving the system by Least Mean Squares:

(15) $$\Delta X={\left(A^{T}\cdot A\right)}^{-1}\cdot A^{T}\cdot B$$

The vector of the estimated positions is updated as in the spherical case and the algorithm is repeated until Δ ***X*** is lower than a pre-determined threshold.

### 2.3. Initialization

The initial estimated test points and the estimated beacons’ positions are randomly selected inside the coverage area. If the selection of the initial estimated variables is very bad, the algorithm cannot converge, so after every update of the solution the feasibility of the new estimated positions is checked; that means that the new positions are inside the coverage area of the beacons.

## 3. Optimal Zones for Test Points

As has been mentioned before, the test points can be located at any place inside the coverage area, but, depending on where they are placed, the measurement errors can affect the final solution. Here a method for determining the best positions inside the coverage area to locate the unknown tests points is developed.

As has been already explained, in order to find the beacons’ location, it is necessary to solve the expression ***A*** · Δ ***X*** = ***B***, where Δ***X*** is a vector that contains all the system unknowns, the study of the covariance of Δ***X*** allows the error in the beacons localization to be minimized:

(16) $$\begin{array}{l} \mathrm{cov}(\Delta X)\;=E[\Delta X\cdot {\Delta X}^{T}]=E\left[({\left(A^{T}A\right)}^{-1}\cdot A^{T}\cdot B)\cdot {\left({\left(A^{T}A\right)}^{-1}\cdot A^{T}\cdot B\right)}^{T}\right]\\ \;\;\;\;\;\;\;\;\;\;\;\;\;\;\;\;\;\;\;\;\;\;\;\;\;\;\;=E[{\left(A^{T}A\right)}^{-1}A^{T}\cdot B\cdot B^{T}\cdot A\cdot {\left(A^{T}\cdot A\right)}^{-1}] \end{array}$$

It is assumed that the covariance of **B** can be expressed as cov(**B**) = I_N_ σ^2^, where *σ_N_* is the error standard deviation in the measurements between the beacons and the test points, and ***I**_N_* is the identity matrix:

(17) $$\mathrm{cov}(\Delta X)=E[{\left(A^{T}A\right)}^{-1}B\cdot B^{T}\cdot (A^{T}\cdot A)\cdot {\left(A^{T}\cdot A\right)}^{-1}]={\left(A^{T}A\right)}^{-1}\mathrm{cov}(B)={\left(A^{T}A\right)}^{-1}I_{N}\sigma^{2}$$

So the function to be minimized to decrease the error in the beacon location and to find the optimal areas of test points is:

(18) $$h=\mathrm{Trace}{\left(A^{T}A\right)}^{-1}$$

**Figure 2.**
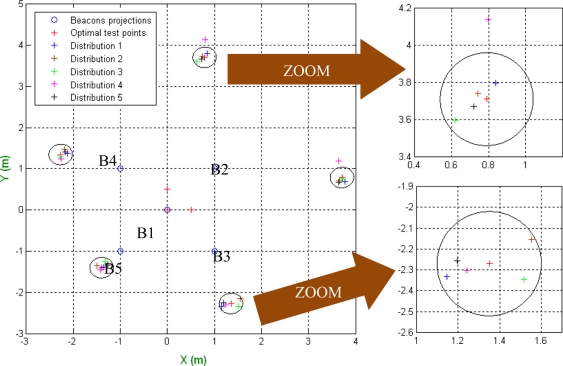
Distribution of the unknown test points around the location of the optimal test points.

Equation (18) allows the determination of the positions where the unknown test points should be placed in order to minimize the PDOP (Position Dilution Of Precision) at the points where the actual beacons are placed—notice that in the calibration process the positions of the beacons are the points to be calculated. Although the function returns the exact positions for the unknown test points, if they are located around these obtained positions, the aforementioned PDOP is near the minimum, so it is not necessary to pose the unknown test points in exactly the right position to obtain good results in the calibration process (an area close to the location is sufficient). As an example of that, the distribution of beacons considered after in the real result is analyzed. Table 1 shows the variation of the PDOP in the location of the beacons, when the test points are moved around of their optimal positions. It can be observed that the differences are less than the 1%. The distribution of the test points used in the test can be observed in Figure 2 where the blue circles are the projection of the beacons in the measurement plane (they are at 3.5 m of height), the red circumference represents a distance of 0.25 m around the optimal test points and the crosses are the positions of the test points for the different configurations for which the PDOP has been calculated.

Notice that to obtain the optimal areas the criterion of minimizing the PDOP in the beacons’ location given a test point configuration is used. Other criteria such as the condition number or the Cramer-Rao limit could have been used, but as has been proven in [9], in that case to pose the beacons in the best areas, the final location of these areas is similar with the three methods. And, regarding the computational load, the calculation of a matrix trace is a best option (only the diagonal elements must be obtained).

In order to find the optimal areas for the simulation and experimental test the function in Equation (18) is minimized using the methods proposed in [10] and [11], which use a non-linear minimization with constraints. The constraints here considered correspond to the limits of the coverage zone of the LPS system. The number of analyzed test-points should be also included in the initialization of the algorithm, It is also necessary to include an initial estimation of the beacons’ positions that is performed using an inverse positioning algorithm with only the three known test points or making a coarse hand-made calibration.

## 4. Simulation Results

The results of tests run under simulation are here presented. The tests are carried out for both, the spherical and the hyperbolic versions of the algorithm.

### 4.1. Spherical Trilateration

In the first simulation, the results obtained for calibrating the beacons by using four random test points, four optimal test points, and eight optimal test points are compared. In this simulation the auto-calibration algorithm is repeated 100 times for different noise situations. The simulation conditions are (all units are considered in meters):

- Beacons placed at: (0,0,3);(2,2,3);(2,−2,3);(−2.2.3);(−2,−2,3)
- The three known test points are at: (0,0,0); (0.5,0,0);(0,0.5,0)
- Four random test points: (−0.7,3.2,0);(−3.5,−1.2,0);(2.5,−3.9,0);(2.9,−2.4,0)
- Four optimal test points: (−4.0, 2.0,0);(−2.8,−1.9,0);(2.9,2.8,0);(0.0,−4.0,0)
- Eight optimal test points: (2.0,−3.3,0); (1.6,4.0,0); (−2.0,3.7,0); (4.0,−1.4,0); (−3.2,−1.9,0); (−3.5,1.5,0); (3.7,2.2,0); (−1.4,−3.4,0)
- The coverage area is *x* = [−4,4] and *y* = [−4,4]
- The noise values are: *σ* = [0.001,0.01,0.04] m.

**Figure 3.**
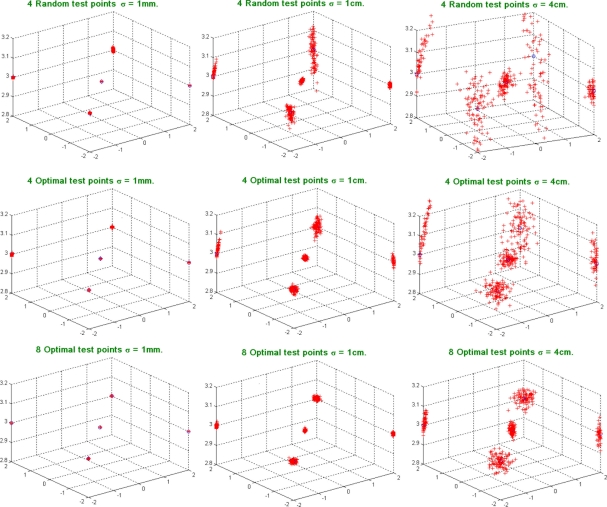
Representation of the 100 auto-calibrations for different noise levels in measurements *σ* = [1 mm, 1 cm, 4 cm], using four random unknown test points; four optimal unknown test points and eight optimal unknown test points (spherical case).

Figure 3 shows how the dispersion in the localization of beacons increases according to the noise level. Also the dispersion in the results is lower when eight optimal test points are used.

**Figure 4.**
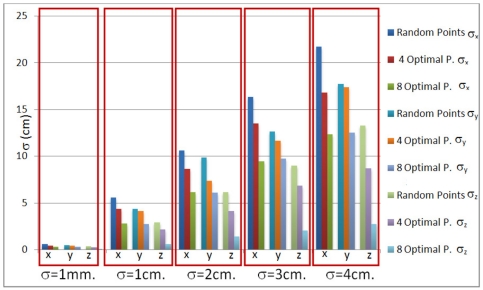
Standard noise deviation in results for 100 simulations (spherical case).

Figure 4 shows the standard deviation in the (*x*, *y*, *z*) components for different noise levels. Notice that the *z* component is the least affected by the noise level; that is because the *z* coordinates of the test points are taken as zero. Also, the results obtained with four optimal test points are similar to those from four random test points, but, when eight optimal test points are used, the results significantly improve.

From Figures 3 and 4 it can be concluded that, in the spherical case, the obtained results are quite similar for optimal and random test points when the noise level in measurements is less than 1 cm. Nevertheless, for higher noise levels it is advisable to use optimal test points.

Table 2 contains the average error (in cm) for every component in the localization of beacons after 100 simulations. In this case the results obtained with the optimal test points are better, but very similar to those with random test points.

### 4.2. Hyperbolic Trilateration

In this case the algorithm based on random test points does not converge for a standard noise deviation in measurements of 4 cm, so a standard noise deviation of 1.5 cm is considered.

For this simulation the four unknown test points are placed at (*z* = 0):

- Random test points: (−2.9,−2.4);(−2.4,0.8);(−1.8 ,−2.4);(−3.9,2.0)
- Four optimal test points: (−0.25,4.0);(4.0,−4.0);(4.0,−0.69);(−4.0,−4.0)
- Eight optimal test points: (−4,−4);(4,0);(−4,4);(0,4);(0,−4);(0,−4);(4,4);(4,−4);(−4,0).
- The noise values are: *σ* = [0.001,0.01,0.15].

**Figure 5.**
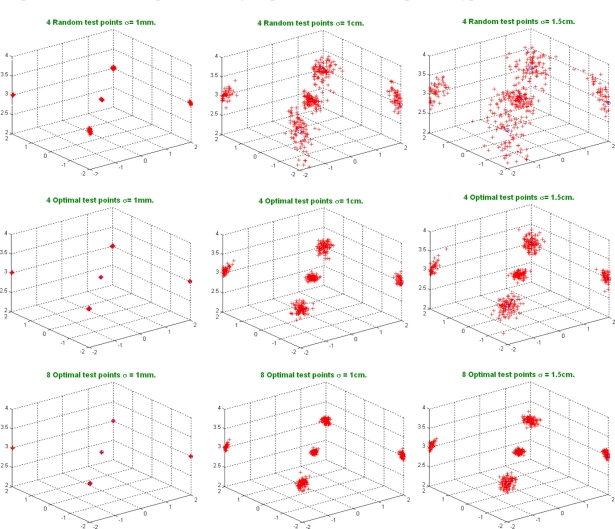
Representation of 100 auto-calibrations for different noise levels in measurements *σ* = [1 mm, 1 cm, 4 cm], using four random unknown test points; four optimal unknown test points and eight optimal unknown test points (hyperbolic case).

Figure 5 shows how the dispersion in results when using random test points is higher than that based on 4 or 8 optimal test points. In fact, for a noise standard deviation higher than 1.5 cm, the algorithm based on random test points does not converge, whereas, when optimal test points are considered, the algorithm still performs.

**Figure 6.**
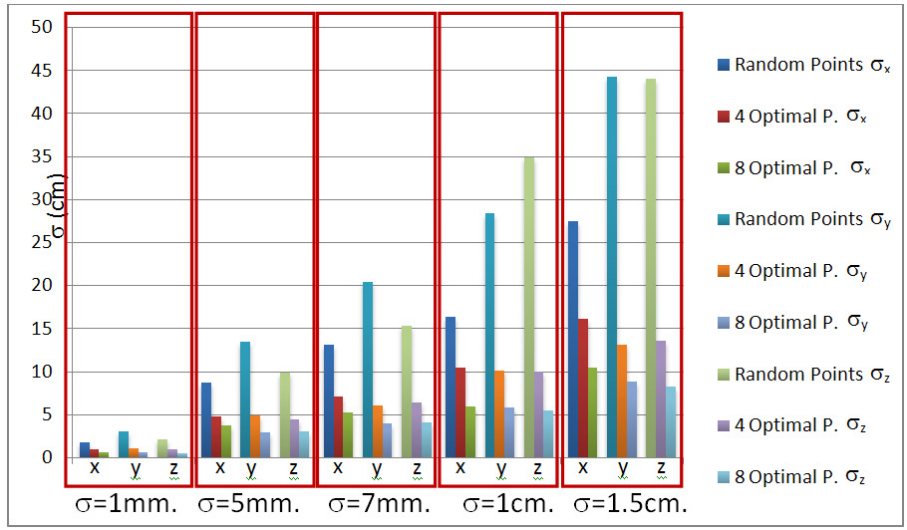
Standard noise deviation in results for 100 simulations (hyperbolic case).

In this case, the standard deviation in the localization of beacons is much lower for optimal test points than for random test points (see Figure 6). As an example, for a standard noise deviation of 1 cm, the standard deviation in results is reduced by half when using four optimal test points, and reduced to a 25% for eight optimal test points.

Table 3 shows the average error (in cm) for every component in the localization of beacons. In this case the results with optimal test points are better, so, if the system uses hyperbolic trilateration for calibration, the test points should be in optimal areas.

**Figure 7.**
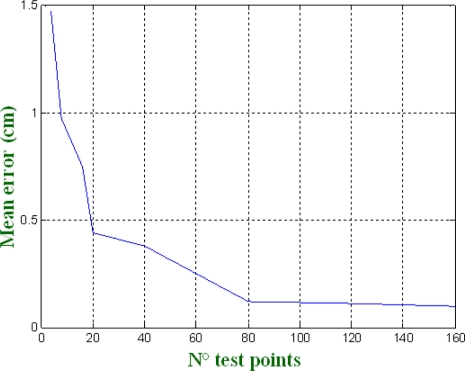
Average error in the beacon localization depending on the number of test points.

By analyzing the results, it can be stated that by increasing the number of test points it is possible to reduce the error in the localization of the beacons. Figure 7 shows the average error in the beacons’ localization $(e=\frac{1}{N}\Sigma_{i=1}^{N}\sqrt{{\left(b_{\mathrm{xi}}-{\hat{b}}_{\mathrm{xi}}\right)}^{2}+{\left(b_{\mathrm{yi}}-{\hat{b}}_{\mathrm{yi}}\right)}^{2}+{\left(b_{\mathrm{zi}}-{\hat{b}}_{\mathrm{zi}}\right)}^{2})}\;$ for different numbers of test points. Notice that, for a certain value (80 in the case analyzed here), the average error improvement is very low and with the increment of the unknown test points the computational load increase dramatically (in this case it has been tested up to 160 test points).

## 5. Experimental Results

**Figure 8.**
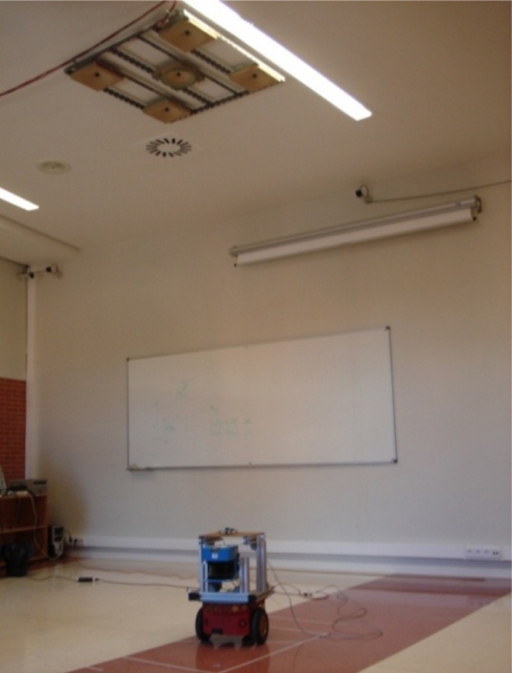
Ultrasonic LPS by GEINTRA used in experimental tests.

Experimental tests have been carried out with the LPS developed by GEINTRA Research Group at the University of Alcalá (see Figure 8). This LPS consists of five ultrasonic beacons placed in the ceiling with a coverage area of 10 m^2^. Descriptions of all the features of this LPS can be found in [12] and [13].

Table 4 shows the position of the beacons for the mentioned GEINTRA LPS (considered in meters). The steps followed to perform the manual calibration were: first a grid of points with a separation of 0.5 m was performed inside the coverage area on the floor using a line laser projector, then the projection of the beacons on the floor was determined using a plumb line and, finally, the height of the beacons was obtained using a ranging laser.

For the rest of the paper it is assumed that the manual calibration is the ground truth and serves as reference to compare with the autocalibration.

Twenty measurements at every point have been done for calibration. Furthermore, the three known test points are placed at these positions (all units in m): (0,0,0.03); (0.5,0,0.03); (0,0.5,0.03). The ranging error standard deviation in the measurements is less than 1 cm [8].

### 5.1. Spherical Trilateration

Two three-dimensional calibrations have been carried out in the LPS. In the first one, four unknown test points have been used, whereas, in the second one, eight unknown test points are considered. The height for all the test points is 3 cm:

- Four unknown test points positions: (−1.5,−1);(−1.5,1.5);(0.5,1.5);(1.5,0.5) m.
- Eight unknown test points positions: (0,−1.5);(−1.5,−1.5);(2,0.5);(−1.5,1.5);(0.5,1.5);(1.5,1.5); (2,−1.5);(−1.5,0) m.

In Table 5 the positions of beacons obtained after calibration are shown (in meters).

**Figure 9.**
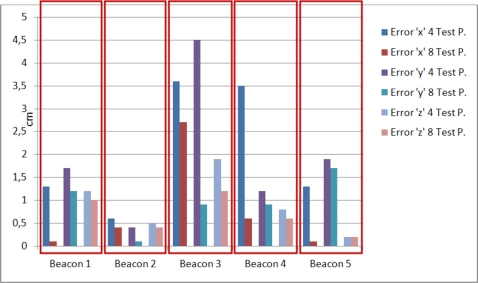
Average absolute error in every component for the calibrated beacons (spherical case).

Figure 9 represents the average absolute error (for 20 measurements) in cm for every component (*x*, *y*, *z*) after calibration; it can be observed that, apart from the beacon 3, this error is below 2 cm. Also the least error is provided by the component *z*.

**Figure 10.**
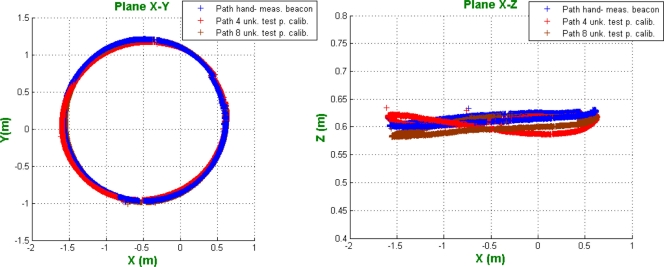
Path obtained using the hand-measured beacon positions and the calibrated beacons (spherical case).

In order to compare the results between localization using a manual calibration of beacons and the beacon auto-calibration, a circular path inside the coverage area has been performed. In Figure 10 the comparison results are shown. Although the handmade calibration has been made carefully, it is not completely error free. The goal of this representation is to show that similar performances can be obtained in both cases.

The positions obtained in the three routes are very similar. In the case of the beacons calibrated with four test points, the mean error in distance is 4.41 cm, whereas, for beacons obtained with eight test points, the error is 3.20 cm.

### 5.2. Hyperbolic Trilateration

The distribution of beacons in the environment is not the most suitable, since they are very close for hyperbolic positioning. This provides a high PDOP in the LPS, so the algorithm does not converge for a calibration in three dimensions. For that reason, only a 2-dimensional calibration is performed and the height of beacons is fixed at 3.5 m. The localizations of the unknown test points for the test are (*z* = 0.03 m):

- Four unknown test points positions: (1,−0.5);(1,1);(−1.5,−0.5);(−1.5,1) m.
- Eight unknown test points positions: (0,−1.5);(−1.5,−1.5);(−1.5,0);(−1.5,1.5);(0.5,1.5);(1.5,1.5); (2,−1.5);(−1.5,0) m.

In Table 6 the position of beacons after calibration are shown (in meters).

**Figure 11.**
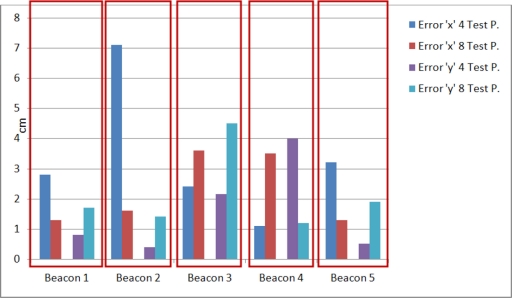
Average absolute error in every component for the calibrated beacons (hyperbolic case).

Figure 11 shows the average absolute error (for 20 measurements) in cm for every component (*x*, *y*) after calibration: these errors are higher than those for the spherical case.

**Figure 12.**
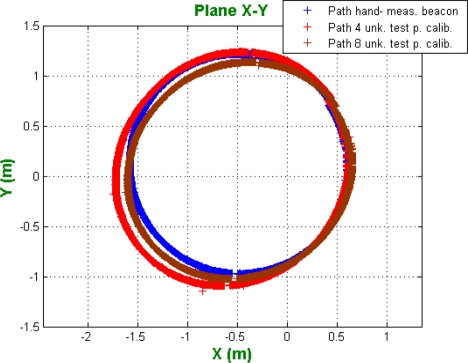
Path obtained using the hand-measured beacon positions and the calibrated beacons (hyperbolic case).

In order to compare the results for localization using the real position of beacons and the beacon auto-calibration, the same path for the mobile robot, as the one commented in the spherical case, is considered. The results can be seen in Figure 12.

Notice that the results are worse than those for spherical trilateration. In fact, the average absolute error in distance using the beacon calibrated with four test points 10.33 cm; and in case of the beacons obtained with eight test points, the error is 7.27 cm

## 6. Conclusions

An algorithm for LPS auto-calibration in two cases, spherical and hyperbolic, has been developed. This algorithm only requires the location of three test points to be known, whereas the position of the other test points can be unknown. The proposed method achieves better performance for spherical trilateration, since this positioning algorithm is more accurate than the hyperbolic one. But, in case of low measurement error (standard deviation less than 5 mm), it is also possible to carry out a satisfactory hyperbolic auto-calibration.

Moreover, how to compute the optimal areas to place the test points in order to achieve improvements in auto-calibration has been described. The use of these areas is important for hyperbolic trilateration. Increasing the number of test points improves the calibration results up to a limit.

## Figures and Tables

**Table 1.** PDOP values in the beacons’ location (B1 to B5) for the different distributions of the unknown test points around the optimal test points.

**PDOP**	**B1**	**B2**	**B3**	**B4**	**B5**

Optimal test points	1.3502	1.3569	1.4205	1.4205	1.4960
Distribution 1	1.3490	1.3623	1.4207	1.4210	1.5010
Distribution 2	1.3512	1.3625	1.4245	1.4235	1.5035
Distribution 3	1.3562	1.3616	1.4313	1.4223	1.4992
Distribution 4	1.3691	1.3801	1.4377	1.4458	1.5079
Distribution 5	1.3537	1.3589	1.4271	1.4243	1.5022

**Table 2.** Average error in the localization of beacons (in cm).

**(cm)**	**4 Random Test Points**			**4 Optimal Test Points**			**8 Optimal Test Points**		

	x	y	z	x	y	z	x	y	z

σ = 1 mm	0.03	0.02	0.02	0.01	0.19	0.01	0.01	0.01	0.01
σ = 1 cm	0.39	0.33	0.33	0.23	0.18	0.06	0.15	0.13	0.06
σ = 2 cm	0.53	0.48	0.45	0.41	0.36	0.12	0.34	0.20	0.09
σ = 3 cm	0.83	0.81	0.79	0.62	0.75	0.31	0.42	0.58	0.12
σ = 4 cm	2.76	0.94	0.88	0.83	0.80	0.66	0.60	0.69	0.19

**Table 3.** Average error in the localization of beacons (in cm).

**(cm)**	**4 Random Test Points**			**4 Optimal Test Points**			**8 Optimal Test Points**		

	x	y	z	x	y	z	x	y	z

σ = 1 mm	0.16	0.33	0.19	0.02	0.04	0.05	0.01	0.02	0.02
σ = 5 mm	0.47	1.04	0.99	0.30	0.63	0.32	0.27	0.18	0.17
σ = 7 mm	0.73	1.35	1.65	0.44	0.90	0.39	0.29	0.24	0.23
σ = 1 cm	1.08	1.86	2.46	0.71	1.09	0.42	0.31	0.29	0.26
σ = 1.5 cm	1.72	2.79	3.93	0.90	1.31	0.75	0.38	0.31	0.30

**Table 4.** Position of beacons in the GEINTRA LPS after a manual calibration.

**(m)**	**Beacon 1**	**Beacon 2**	**Beacon 3**	**Beacon 4**	**Beacon 5**

Coord. X	0.122	0.513	0.515	−0.24	−0.242
Coord. Y	0.224	0.56	−0.1	−0.11	0.56
Coord. Z	3.5	3.5	3.5	3.5	3.5

**Table 5.** Position of beacons after calibration (spherical case).

**(m)**	**4 Unknown Test Points**					**8 Unknown Test Points**				

	B1	B2	B3	B4	B5	B1	B2	B3	B4	B5

Coord. X	0.135	0.507	0.551	−0.205	−0.229	0.122	0.504	0.542	−0.234	−0.242
Coord. Y	0.207	0.556	−0.145	−0.122	0.541	0.212	0.561	−0.109	−0.119	0.534
Coord. Z	3.488	3.495	3.481	3.492	3.498	3.487	3.495	3.488	3.489	3.496

**Table 6.** Position of beacons after calibration (hyperbolic case).

**(m)**	**4 Unknown Test Points**					**8 Unknown Test Points**				

	B1	B2	B3	B4	B5	B1	B2	B3	B4	B5

Coord. X	0.094	0.442	0.491	−0.229	−0.274	0.135	0.497	0.551	−0.205	−0.229
Coord. Y	0.233	0.564	−0.079	−0.070	0.554	0.207	0.546	−0.145	−0.122	0.541
Coord. Z	3.5	3.5	3.5	3.5	3.5	3.5	3.5	3.5	3.5	3.5
